# Crystal structure of *mer*-tris­{2,6-di­fluoro-3-[5-(2-fluoro­phen­yl)pyridin-2-yl-κ*N*]pyridin-4-yl-κ*C* ^4^}iridium(III) di­chloro­methane hemisolvate *n*-hexane hemisolvate

**DOI:** 10.1107/S2056989017016759

**Published:** 2017-11-28

**Authors:** Youngjin Kang, Ki-Min Park, Jinho Kim

**Affiliations:** a Division of Science Education & Department of Chemistry, Kangwon National University, Chuncheon 24341, Republic of Korea; b Research Institute of Natural Science, Gyeongsang National University, Jinju 52828, Republic of Korea

**Keywords:** crystal structure, iridium(III) complex, *C*,*N*-chelating ligand, *mer*-C_3_N_3_ coordination set, hydrogen bonds, C—F⋯π inter­actions

## Abstract

The asymmetric unit of the title compound, [Ir(C_17_H_11_F_2_N_2_)_3_]·0.5CH_3_(CH_2_)_4_CH_3_·0.5CH_2_Cl_2_, comprises one Ir^III^ atom, three 2,6-di­fluoro-3-[5-(2-fluoro­phen­yl)pyridin-2-yl]pyridin-4-yl ligands and half each of an *n*-hexane and a di­chloro­methane solvent mol­ecule located about crystallographic inversion centres. The Ir^III^ atom displays a distorted octa­hedral coordination geometry, having three *C*,*N*-chelating 2,6-di­fluoro-3-[5-(2-fluoro­phen­yl)pyridin-2-yl]pyridin-4-yl ligands arranged in a meridional manner. The Ir^III^ ion lies almost in the equatorial plane [deviation = 0.0069 (15) Å]. The average distance [2.041 (3) Å] of Ir—C bonds is slightly shorter than that [2.076 (3) Å] of Ir—N bonds. A variety of intra- and inter­molecular C—H⋯F and C—H⋯π hydrogen bonds, as well as inter­molecular C—F⋯π inter­actions, contribute to the stabilization of the mol­ecular and crystal structures, and result in the formation of a two-dimensional network parallel to the *ab* plane. No inter­actions between *n*-hexane solvent mol­ecules and the other components in the title compound are observed.

## Abstract 2

Iridium(III) complexes based on 2,3′-bi­pyridine ligands are known to exhibit strong emission from blue to green that makes them of inter­est for organic light-emitting diodes and organic lighting uses. In the title compound, the Ir^III^ ion adopts a distorted octa­hedral coordination environment defined by three *C*,*N*-chelating 2,6-di­fluoro-3-[5-(2-fluoro­phen­yl)pyridin-2-yl]pyridin-4-yl ligands in a meridional manner. In the crystal, inter­molecular C—H⋯F and C—H⋯π hydrogen bonds, as well as inter­molecular C—F⋯π inter­actions, are present, leading to a two-dimensional network.

## Chemical context   

Phospho­rescent iridium(III) complexes are considered to be excellent candidates for triplet emitters in phospho­rescent organic light-emitting diodes (PHOLEDs) because of their high efficiency and high stability (Cho *et al.*, 2016 ▸). In partic­ular, iridium(III) complexes with *C*,*N-*chelating 2,3′-bi­pyridine ligands have recently attracted much attention because of their deep-blue emission and easy tuning emission energy upon ligand substitution (Kim *et al.*, 2017 ▸). However, many studies of the crystal structures of bi­pyridine-based iridium(III) derivatives are focused on the different substituents of the C-coordinating pyridine ring (Lee *et al.*, 2014 ▸). Examples of iridium(III) complexes with substituents on the N-coordin­ating pyridine ring are relatively rare compared to those of C-coordination pyridine-functionalized iridium(III) complexes (Lee *et al.*, 2016 ▸; Oh *et al.*, 2013 ▸). Herein, we report the result of our investigation of the crystal structure of an iridium(III) complex with an *o*-tolyl group on the N-coordin­ating pyridine ring.

## Structural commentary   

**Figure 1:**
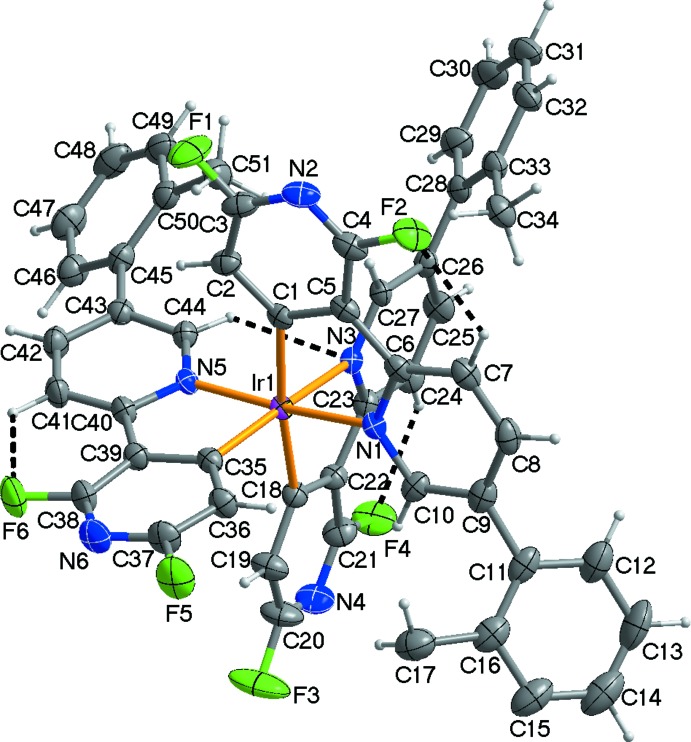
View of the mol­ecular structure of the title compound, showing the atom-numbering scheme. Displacement ellipsoids are drawn at the 50% probability level; dashed lines represent intra­molecular C—H⋯F and C—H⋯N hydrogen bonds. The *n*-hexane and di­chloro­methane solvent mol­ecules are not shown for clarity.

The mol­ecular structure of the title compound is shown in Fig. 1 ▸. The asymmetric unit consists of one Ir^III^ atom, three 2,6-di­fluoro-3-[5-(2-fluoro­phen­yl)pyridin-2-yl]pyridin-4-yl ligands, and half each of the *n*-hexane and di­chloro­methane solvent mol­ecules located about crystallographic inversion centres. The Ir^III^ atom is six-coordinated by three *C*,*N*-chelating 2,6-di­fluoro-3-[5-(2-fluoro­phen­yl)pyridin-2-yl]pyrid­in-4-yl ligands, forming a distorted octa­hedral coordination sphere due to narrow ligand bite angles, which range from 78.49 (12) to 80.32 (12)° (Table 1 ▸). The pyridyl N atoms of the three ligands are arranged in a *mer*-configuration around the octa­hedral Ir^III^ ion (Fig. 1 ▸). The equatorial plane is defined by the N1/C1/N5/C18 atoms, the mean deviation from the least-squares plane being 0.0585 (14) Å. The Ir^III^ ion lies almost in the equatorial plane with a deviation of 0.0069 (15) Å. As listed in Table 1 ▸, the Ir—C and Ir—N bond lengths in the title compound are within the ranges reported for similar Ir^III^ compounds, for example, *mer*-[tris­[2′,6′-di­fluoro-2,3′-bipyri­dinato-*k* ^2^ *C* ^4′^,*N*]iridium(III)] (Jung *et al.*, 2012 ▸). The average length [2.041 (3) Å] of the Ir—C bonds is slightly shorter than that [2.076 (3) Å] of the Ir—N bonds because of back bonding between the metal and an anionic C atom of the ligand. Within the ligands, the terminal pyridine rings are tilted slightly by 7.2 (2), 6.74 (19), and 6.29 (18)°, respectively, to the N1-, N3-, and N5-containing central pyridine rings, indicating that effective π conjugations of the two pyridine rings occur in the ligands. The terminal phenyl rings, however, are tilted by 51.79 (13), 46.74 (11), and 40.50 (12)° with respect to N1-, N3-, and N5-containing central pyridine rings, respectively. The mol­ecular structure of the Ir^III^ complex is stabilized by weak intra­molecular C—H⋯F and C—H⋯N hydrogen bonds (Table 2 ▸, shown as dashed lines in Fig. 1 ▸).
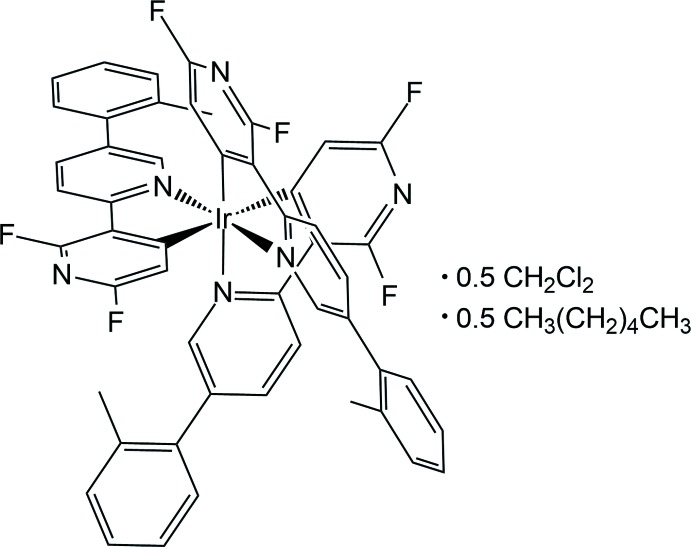


## Supra­molecular features   

**Figure 2:**
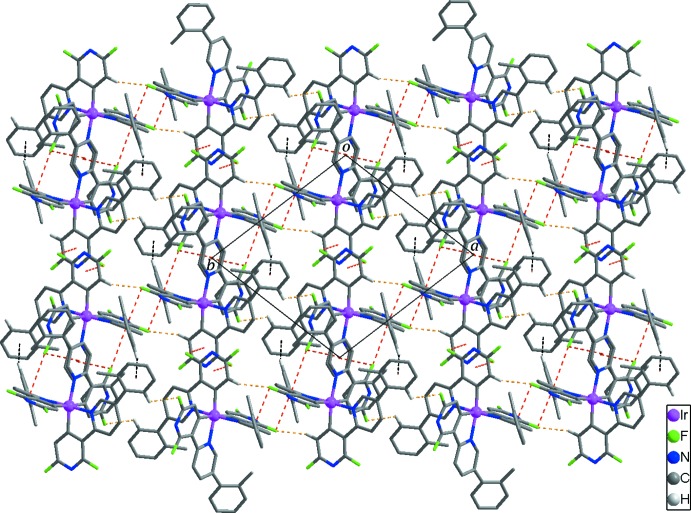
The two-dimensional supra­molecular network formed through inter­molecular C—H⋯F hydrogen bonds (yellow dashed lines) and C—H⋯π (black dashed lines) and inter­molecular C—F⋯π inter­actions (red dashed lines). H atoms not involved in inter­molecular inter­actions and the lattice solvent mol­ecules are not shown for clarity.

Inter­molecular C—H⋯F hydrogen bonds (Table 2 ▸, yellow dashed lines in Fig. 2 ▸) between adjacent Ir^III^ complexes lead to the formation of one-dimensional chains propagating along the [110] direction. These chains are further inter­linked by C13—H⋯π inter­actions (Table 2 ▸, black dashed lines in Fig. 2 ▸), resulting in the formation of a two-dimensional supra­molecular network extending parallel to the *ab* plane. In addition, weak inter­molecular C—F⋯π inter­actions [F2⋯*Cg*1^i^ = 3.268 (3) Å; F4⋯*Cg*1^iii^ = 3.411 (3) Å; F4⋯*Cg*2^iii^ = 3.387 (3) Å; F6⋯*Cg*5^iv^ = 3.291 (3) Å; *Cg*1, *Cg*2, and *Cg*5 are the centroids of the N1/C6–C10, N3/C23–C27, and N6/C35–C39 rings, respectively; symmetry codes: (i) −*x* + 2, −*y* + 1, −z + 1; (iii) −*x* + 2, −*y* + 2, −z + 1; (iv) −*x* + 1, −*y* + 1, −z + 1] contribute to the stabilization of the crystal structure. Inter­molecular C55—H⋯π inter­actions (Table 2 ▸) between the Ir^III^ complexes and the disordered di­chloro­methane solvent mol­ecules also occur in the crystal structure of the title compound (not shown in Fig. 2 ▸).

No inter­actions between the *n*-hexane solvent mol­ecules and the other components of the title compound are observed.

## Synthesis and crystallization   

The title complex was synthesized according to a previous report (Lee *et al.*, 2017 ▸). Slow evaporation from a di­chloro­methane/hexane solution afforded yellow crystals suitable for X-ray crystallography analysis. ^1^H NMR(400 MHz, CD_2_Cl_2_): δ 8.89 (*dd*, *J* = 6.2, 1.4 Hz, 1H), 8.30 (*dd*, *J* = 8.8, 1.0 Hz, 1H), 8.27–8.21 (*m*, 2H), 8.01 (*d*, *J* = 2.0 Hz, 1H), 7.91 (d, *J* = 1.2 Hz, 1H), 7.76–7.70 (*m*, 3H), 7.59 (*d*, *J* = 1.6 Hz, 1H), 7.2–6.95 (*m*, 12H), 6.31 (*t*, *J* = 3.2 Hz, 1H), 5.94 (*t*, *J* = 2.4 Hz, 1H), 5.83 (*t*, *J* = 2.0 Hz, 1H), 1.94 (*s*, 3H), 1.93 (*s*, 3H), 1.79 (*s*, 3H).

## Refinement   

Crystal data, data collection and structure refinement details are summarized in Table 3 ▸. The di­chloro­methane mol­ecule is disordered over two sets of sites about an inversion centre with equal occupancy. The C—Cl bond lengths were restrained using the DFIX instructions in *SHELXL2014/7* (Sheldrick, 2015 ▸). The anisotropic displacement ellipsoid of a chloride atom (Cl1) in the disordered di­chloro­methane solvent mol­ecule was very elongated and therefore an ISOR restraint was applied for this atom (McArdle, 1995 ▸; Sheldrick, 2008 ▸). All H atoms were positioned geometrically and refined as riding: C—H = 0.95 Å for C*sp* ^2^—H, 0.99 Å for methyl­ene C—H, and 0.98 Å for methyl C—H, with *U*_iso_(H) = 1.2–1.5*U*_eq_(C).

## Supplementary Material

Crystal structure: contains datablock(s) I, New_Global_Publ_Block. DOI: 10.1107/S2056989017016759/hg5501sup1.cif

Structure factors: contains datablock(s) I. DOI: 10.1107/S2056989017016759/hg5501Isup2.hkl

CCDC reference: 1586829

Additional supporting information:  crystallographic information; 3D view; checkCIF report

## Figures and Tables

**Table 1:** Selected geometric parameters (Å, °)

Ir1—C35	1.991 (3)	Ir1—C1	2.061 (3)
Ir1—N5	2.030 (3)	Ir1—C18	2.070 (3)
Ir1—N1	2.056 (3)	Ir1—N3	2.143 (3)
			
C35—Ir1—N5	80.32 (12)	N1—Ir1—C18	97.10 (12)
C35—Ir1—N1	98.62 (12)	C1—Ir1—C18	173.39 (11)
N5—Ir1—N1	174.15 (10)	C35—Ir1—N3	170.98 (11)
C35—Ir1—C1	92.12 (12)	N5—Ir1—N3	94.39 (10)
N5—Ir1—C1	94.75 (12)	N1—Ir1—N3	87.37 (10)
N1—Ir1—C1	79.51 (12)	C1—Ir1—N3	95.61 (11)
C35—Ir1—C18	94.00 (13)	C18—Ir1—N3	78.49 (12)
N5—Ir1—C18	88.72 (11)		

**Table 2:** Hydrogen-bond geometry (Å, °) *Cg*3, *Cg*4 and *Cg*6 are the centroids of the N4/C18–C21, N5/C40–C44, and C45–C50 rings, respectively.

*D*—H⋯*A*	*D*—H	H⋯*A*	*D*⋯*A*	*D*—H⋯*A*
C7—H7⋯F2	0.95	2.29	2.895 (5)	121
C24—H24⋯F4	0.95	2.22	2.851 (4)	123
C36—H36⋯F2^i^	0.95	2.41	3.245 (4)	146
C41—H41⋯F6	0.95	2.32	2.917 (4)	121
C44—H44⋯N3	0.95	2.50	3.112 (4)	122
C46—H46⋯F3^ii^	0.95	2.50	3.067 (5)	119
C13—H13⋯*Cg*6^iii^	0.95	2.98	3.777 (7)	142
C55—H55*A*⋯*Cg*4^ii^	0.99	2.96	3.326 (9)	103
C55—H55*B*⋯*Cg*3^ii^	0.99	3.00	3.718 (10)	131
C55—H55*B*⋯*Cg*4^ii^	0.99	2.78	3.326 (10)	116

Symmetry codes: (i) 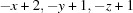; (ii) 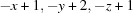; (iii) 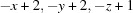.

**Table 3:** Experimental details

Crystal data	
Chemical formula	[Ir(C_17_H_11_F_2_N_2_)_3_]·0.5C_6_H_14_·0.5CH_2_Cl_2_
*M*_r_	1121.58
Crystal system, space group	Triclinic, *P* 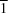
Temperature (K)	173
*a*, *b*, *c* (Å)	12.5753 (2), 14.4054 (3), 14.8668 (3)
α, β, γ (°)	117.0678 (5), 101.9336 (6), 97.2102 (6)
*V* (Å^3^)	2270.58 (8)
*Z*	2
Radiation type	Mo *K*α
μ (mm^−1^)	3.07
Crystal size (mm)	0.42 × 0.23 × 0.21

Data collection	
Diffractometer	Bruker APEXII CCD
Absorption correction	Multi-scan (*SADABS*; Bruker, 2014 ▸)
*T*_min_, *T*_max_	0.532, 0.746
No. of measured, independent and observed [*I* > 2σ(*I*)] reflections	40374, 11187, 10233
*R*_int_	0.035
(sin θ/λ)_max_ (Å^−1^)	0.666

Refinement	
*R*[*F* ^2^ > 2σ(*F* ^2^)], *wR*(*F* ^2^), *S*	0.030, 0.080, 1.04
No. of reflections	11187
No. of parameters	625
No. of restraints	16
H-atom treatment	H-atom parameters constrained
Δρ_max_, Δρ_min_ (e Å^−3^)	2.38, −2.06

Computer programs: *APEX2* and *SAINT* (Bruker, 2014 ▸), *SHELXS97* and *SHELXTL* (Sheldrick, 2008 ▸), *SHELXL2014* (Sheldrick, 2015 ▸), *DIAMOND* (Brandenburg, 2010 ▸) and *publCIF* (Westrip, 2010 ▸).

## supplementary crystallographic information

### Crystal data

**Table:**

[Ir(C_17_H_11_F_2_N_2_)_3_]·0.5C_6_H_14_·0.5CH_2_Cl_2_	*Z* = 2
*M**_r_* = 1121.58	*F*(000) = 1116
Triclinic, *P*1	*D*_x_ = 1.640 Mg m^−^^3^
*a* = 12.5753 (2) Å	Mo *K*α radiation, λ = 0.71073 Å
*b* = 14.4054 (3) Å	Cell parameters from 9893 reflections
*c* = 14.8668 (3) Å	θ = 2.3–28.2°
α = 117.0678 (5)°	µ = 3.07 mm^−^^1^
β = 101.9336 (6)°	*T* = 173 K
γ = 97.2102 (6)°	Block, yellow
*V* = 2270.58 (8) Å^3^	0.42 × 0.23 × 0.21 mm

### Data collection

**Table:**

Bruker APEXII CCD diffractometer	10233 reflections with *I* > 2σ(*I*)
φ and ω scans	*R*_int_ = 0.035
Absorption correction: multi-scan (SADABS; Bruker, 2014)	θ_max_ = 28.3°, θ_min_ = 1.6°
*T*_min_ = 0.532, *T*_max_ = 0.746	*h* = −16→16
40374 measured reflections	*k* = −19→19
11187 independent reflections	*l* = −18→19

### Refinement

**Table:**

Refinement on *F*^2^	16 restraints
Least-squares matrix: full	Hydrogen site location: inferred from neighbouring sites
*R*[*F*^2^ > 2σ(*F*^2^)] = 0.030	H-atom parameters constrained
*wR*(*F*^2^) = 0.080	*w* = 1/[σ^2^(*F*_o_^2^) + (0.047*P*)^2^ + 1.7767*P*] where *P* = (*F*_o_^2^ + 2*F*_c_^2^)/3
*S* = 1.04	(Δ/σ)_max_ = 0.003
11187 reflections	Δρ_max_ = 2.38 e Å^−^^3^
625 parameters	Δρ_min_ = −2.06 e Å^−^^3^

### Special details

**Table:**

Geometry. All esds (except the esd in the dihedral angle between two l.s. planes) are estimated using the full covariance matrix. The cell esds are taken into account individually in the estimation of esds in distances, angles and torsion angles; correlations between esds in cell parameters are only used when they are defined by crystal symmetry. An approximate (isotropic) treatment of cell esds is used for estimating esds involving l.s. planes.

### Fractional atomic coordinates and isotropic or equivalent isotropic displacement parameters (Å^2^)

**Table:**

	*x*	*y*	*z*	*U*_iso_*/*U*_eq_	Occ. (<1)
Ir1	0.80627 (2)	0.75047 (2)	0.53831 (2)	0.01827 (4)	
F1	0.8673 (2)	0.6415 (2)	0.8511 (2)	0.0543 (7)	
F2	1.12093 (19)	0.59363 (19)	0.6739 (2)	0.0429 (6)	
N1	0.9274 (2)	0.6888 (2)	0.4727 (2)	0.0208 (5)	
N2	0.9932 (3)	0.6203 (2)	0.7616 (3)	0.0340 (7)	
C1	0.8725 (2)	0.6929 (2)	0.6358 (3)	0.0212 (6)	
C2	0.8363 (3)	0.6866 (3)	0.7161 (3)	0.0276 (7)	
H2	0.7702	0.7072	0.7304	0.033*	
C3	0.8993 (3)	0.6498 (3)	0.7734 (3)	0.0341 (8)	
C4	1.0248 (3)	0.6251 (3)	0.6851 (3)	0.0298 (7)	
C5	0.9706 (3)	0.6581 (2)	0.6176 (3)	0.0232 (6)	
C6	1.0025 (2)	0.6582 (2)	0.5291 (3)	0.0229 (6)	
C7	1.0955 (3)	0.6311 (3)	0.4960 (3)	0.0303 (7)	
H7	1.1495	0.6127	0.5356	0.036*	
C8	1.1101 (3)	0.6307 (3)	0.4064 (3)	0.0330 (8)	
H8	1.1749	0.6136	0.3859	0.040*	
C9	1.0306 (3)	0.6551 (3)	0.3459 (3)	0.0300 (7)	
C10	0.9416 (3)	0.6851 (2)	0.3839 (3)	0.0252 (7)	
H10	0.8873	0.7041	0.3450	0.030*	
C11	1.0395 (3)	0.6562 (3)	0.2480 (3)	0.0339 (8)	
C12	1.1385 (4)	0.7141 (4)	0.2506 (4)	0.0489 (11)	
H12	1.2007	0.7477	0.3130	0.059*	
C13	1.1479 (5)	0.7235 (4)	0.1634 (5)	0.0611 (14)	
H13	1.2159	0.7634	0.1662	0.073*	
C14	1.0573 (5)	0.6742 (4)	0.0724 (4)	0.0597 (14)	
H14	1.0625	0.6816	0.0131	0.072*	
C15	0.9605 (5)	0.6148 (4)	0.0677 (4)	0.0510 (11)	
H15	0.8996	0.5801	0.0042	0.061*	
C16	0.9490 (4)	0.6041 (3)	0.1545 (3)	0.0388 (9)	
C17	0.8413 (4)	0.5336 (4)	0.1405 (4)	0.0510 (11)	
H17A	0.8564	0.4994	0.1840	0.077*	
H17B	0.8090	0.4780	0.0659	0.077*	
H17C	0.7882	0.5775	0.1625	0.077*	
F3	0.5772 (3)	0.7962 (2)	0.2111 (2)	0.0757 (10)	
F4	0.8327 (2)	1.08740 (18)	0.4761 (2)	0.0466 (6)	
N3	0.9257 (2)	0.9044 (2)	0.6254 (2)	0.0202 (5)	
N4	0.7040 (3)	0.9417 (3)	0.3469 (3)	0.0402 (8)	
C18	0.7582 (3)	0.8195 (3)	0.4460 (3)	0.0242 (6)	
C19	0.6756 (3)	0.7747 (3)	0.3490 (3)	0.0350 (8)	
H19	0.6349	0.7013	0.3136	0.042*	
C20	0.6547 (4)	0.8392 (4)	0.3060 (3)	0.0440 (10)	
C21	0.7826 (3)	0.9830 (3)	0.4368 (3)	0.0321 (8)	
C22	0.8172 (3)	0.9295 (3)	0.4913 (3)	0.0236 (6)	
C23	0.9103 (3)	0.9743 (2)	0.5882 (3)	0.0229 (6)	
C24	0.9824 (3)	1.0773 (3)	0.6421 (3)	0.0299 (7)	
H24	0.9710	1.1270	0.6179	0.036*	
C25	1.0702 (3)	1.1068 (3)	0.7304 (3)	0.0307 (7)	
H25	1.1189	1.1771	0.7667	0.037*	
C26	1.0887 (3)	1.0351 (3)	0.7673 (3)	0.0241 (6)	
C27	1.0117 (2)	0.9351 (2)	0.7118 (3)	0.0212 (6)	
H27	1.0204	0.8854	0.7365	0.025*	
C28	1.1851 (3)	1.0651 (3)	0.8603 (3)	0.0259 (7)	
C29	1.2073 (3)	1.1663 (3)	0.9495 (3)	0.0377 (8)	
H29	1.1586	1.2118	0.9500	0.045*	
C30	1.2990 (4)	1.2017 (3)	1.0375 (3)	0.0457 (10)	
H30	1.3132	1.2709	1.0976	0.055*	
C31	1.3691 (3)	1.1359 (4)	1.0369 (3)	0.0459 (11)	
H31	1.4327	1.1599	1.0964	0.055*	
C32	1.3476 (3)	1.0351 (3)	0.9502 (3)	0.0380 (9)	
H32	1.3965	0.9903	0.9516	0.046*	
C33	1.2560 (3)	0.9967 (3)	0.8603 (3)	0.0293 (7)	
C34	1.2406 (3)	0.8875 (3)	0.7682 (3)	0.0349 (8)	
H34A	1.2296	0.8922	0.7033	0.052*	
H34B	1.1748	0.8377	0.7613	0.052*	
H34C	1.3075	0.8611	0.7796	0.052*	
F5	0.5676 (2)	0.34823 (18)	0.2318 (2)	0.0532 (7)	
F6	0.38409 (17)	0.55554 (19)	0.4386 (2)	0.0466 (6)	
N5	0.6894 (2)	0.8035 (2)	0.6117 (2)	0.0210 (5)	
N6	0.4774 (3)	0.4534 (3)	0.3367 (3)	0.0386 (8)	
C35	0.6786 (3)	0.6197 (3)	0.4518 (3)	0.0232 (6)	
C36	0.6739 (3)	0.5210 (3)	0.3648 (3)	0.0298 (7)	
H36	0.7383	0.5075	0.3418	0.036*	
C37	0.5732 (3)	0.4448 (3)	0.3142 (3)	0.0356 (8)	
C38	0.4829 (3)	0.5465 (3)	0.4172 (3)	0.0324 (8)	
C39	0.5777 (3)	0.6319 (3)	0.4795 (3)	0.0255 (7)	
C40	0.5854 (3)	0.7342 (3)	0.5712 (3)	0.0246 (6)	
C41	0.5035 (3)	0.7659 (3)	0.6217 (3)	0.0325 (8)	
H41	0.4299	0.7199	0.5929	0.039*	
C42	0.5293 (3)	0.8633 (3)	0.7127 (3)	0.0339 (8)	
H42	0.4734	0.8836	0.7469	0.041*	
C43	0.6366 (3)	0.9335 (3)	0.7561 (3)	0.0256 (7)	
C44	0.7122 (3)	0.8998 (3)	0.6997 (3)	0.0243 (6)	
H44	0.7842	0.9473	0.7245	0.029*	
C45	0.6669 (3)	1.0390 (3)	0.8546 (3)	0.0288 (7)	
C46	0.5870 (3)	1.0993 (3)	0.8704 (3)	0.0356 (8)	
H46	0.5167	1.0726	0.8173	0.043*	
C47	0.6081 (4)	1.1971 (3)	0.9618 (3)	0.0428 (9)	
H47	0.5525	1.2367	0.9713	0.051*	
C48	0.7093 (4)	1.2364 (3)	1.0383 (3)	0.0456 (10)	
H48	0.7247	1.3038	1.1009	0.055*	
C49	0.7893 (3)	1.1773 (3)	1.0239 (3)	0.0385 (9)	
H49	0.8592	1.2055	1.0778	0.046*	
C50	0.7712 (3)	1.0787 (3)	0.9339 (3)	0.0311 (7)	
C51	0.8620 (3)	1.0185 (4)	0.9291 (3)	0.0417 (9)	
H51A	0.8275	0.9409	0.8916	0.063*	
H51B	0.9071	1.0410	1.0011	0.063*	
H51C	0.9104	1.0347	0.8915	0.063*	
C52	0.3642 (9)	0.5534 (10)	0.1629 (11)	0.164 (5)	
H52A	0.3244	0.5125	0.1889	0.246*	
H52B	0.4233	0.6141	0.2223	0.246*	
H52C	0.3111	0.5806	0.1289	0.246*	
C53	0.4179 (10)	0.4784 (9)	0.0806 (10)	0.143 (4)	
H53A	0.4697	0.4498	0.1153	0.171*	
H53B	0.3576	0.4164	0.0220	0.171*	
C54	0.4771 (10)	0.5289 (7)	0.0381 (9)	0.140 (4)	
H54A	0.4255	0.5619	0.0091	0.168*	
H54C	0.5386	0.5887	0.0977	0.168*	
C55	0.4858 (8)	1.0429 (5)	0.4679 (6)	0.070 (3)	0.5
H55A	0.5258	1.0535	0.4212	0.084*	0.5
H55B	0.4281	1.0844	0.4747	0.084*	0.5
Cl1	0.4193 (4)	0.9067 (4)	0.4086 (4)	0.245 (2)	

### Atomic displacement parameters (Å^2^)

**Table:**

	*U*^11^	*U*^22^	*U*^33^	*U*^12^	*U*^13^	*U*^23^
Ir1	0.01779 (6)	0.01590 (6)	0.02123 (7)	0.00411 (4)	0.00436 (4)	0.00994 (5)
F1	0.0766 (18)	0.0687 (17)	0.0456 (15)	0.0301 (14)	0.0258 (14)	0.0449 (14)
F2	0.0406 (12)	0.0478 (13)	0.0472 (14)	0.0263 (10)	0.0083 (10)	0.0272 (12)
N1	0.0205 (12)	0.0147 (12)	0.0255 (14)	0.0042 (9)	0.0059 (10)	0.0090 (11)
N2	0.0455 (17)	0.0254 (15)	0.0286 (16)	0.0110 (13)	0.0018 (14)	0.0147 (13)
C1	0.0210 (13)	0.0137 (13)	0.0243 (16)	−0.0001 (11)	0.0012 (12)	0.0092 (12)
C2	0.0326 (17)	0.0247 (16)	0.0260 (17)	0.0048 (13)	0.0065 (14)	0.0144 (15)
C3	0.049 (2)	0.0285 (18)	0.0264 (18)	0.0083 (16)	0.0070 (16)	0.0173 (16)
C4	0.0316 (17)	0.0196 (15)	0.0303 (18)	0.0082 (13)	−0.0005 (14)	0.0096 (14)
C5	0.0233 (14)	0.0149 (14)	0.0267 (16)	0.0040 (11)	0.0009 (12)	0.0096 (13)
C6	0.0216 (14)	0.0151 (14)	0.0260 (16)	0.0038 (11)	0.0032 (12)	0.0071 (13)
C7	0.0259 (15)	0.0233 (16)	0.036 (2)	0.0095 (13)	0.0059 (14)	0.0102 (15)
C8	0.0266 (16)	0.0265 (17)	0.041 (2)	0.0090 (13)	0.0154 (15)	0.0103 (16)
C9	0.0368 (18)	0.0184 (15)	0.0346 (19)	0.0066 (13)	0.0173 (15)	0.0103 (15)
C10	0.0331 (16)	0.0166 (14)	0.0278 (17)	0.0061 (12)	0.0122 (14)	0.0114 (13)
C11	0.050 (2)	0.0235 (16)	0.040 (2)	0.0162 (15)	0.0278 (18)	0.0172 (16)
C12	0.057 (3)	0.042 (2)	0.053 (3)	0.009 (2)	0.032 (2)	0.022 (2)
C13	0.071 (3)	0.057 (3)	0.083 (4)	0.018 (3)	0.053 (3)	0.044 (3)
C14	0.092 (4)	0.056 (3)	0.061 (3)	0.035 (3)	0.051 (3)	0.038 (3)
C15	0.079 (3)	0.048 (3)	0.047 (3)	0.029 (2)	0.034 (2)	0.030 (2)
C16	0.057 (2)	0.0300 (19)	0.040 (2)	0.0194 (17)	0.0244 (19)	0.0198 (18)
C17	0.062 (3)	0.052 (3)	0.040 (2)	0.010 (2)	0.013 (2)	0.026 (2)
F3	0.092 (2)	0.0570 (17)	0.0537 (18)	0.0057 (16)	−0.0304 (16)	0.0328 (15)
F4	0.0643 (15)	0.0293 (11)	0.0506 (15)	0.0084 (10)	0.0053 (12)	0.0290 (11)
N3	0.0202 (11)	0.0174 (12)	0.0256 (14)	0.0064 (9)	0.0090 (10)	0.0115 (11)
N4	0.0490 (19)	0.0394 (18)	0.0415 (19)	0.0159 (15)	0.0069 (16)	0.0291 (17)
C18	0.0242 (15)	0.0263 (16)	0.0272 (17)	0.0100 (12)	0.0090 (13)	0.0159 (14)
C19	0.0382 (19)	0.0275 (18)	0.0309 (19)	0.0045 (14)	−0.0030 (15)	0.0142 (16)
C20	0.051 (2)	0.044 (2)	0.034 (2)	0.0133 (18)	−0.0040 (18)	0.0230 (19)
C21	0.0415 (19)	0.0271 (17)	0.036 (2)	0.0128 (15)	0.0114 (16)	0.0215 (16)
C22	0.0267 (15)	0.0240 (15)	0.0264 (17)	0.0110 (12)	0.0096 (13)	0.0156 (14)
C23	0.0246 (14)	0.0194 (14)	0.0286 (17)	0.0084 (12)	0.0109 (13)	0.0132 (14)
C24	0.0330 (17)	0.0217 (16)	0.039 (2)	0.0084 (13)	0.0110 (15)	0.0181 (16)
C25	0.0341 (17)	0.0158 (15)	0.035 (2)	0.0017 (13)	0.0069 (15)	0.0095 (15)
C26	0.0235 (14)	0.0197 (15)	0.0266 (17)	0.0031 (12)	0.0092 (13)	0.0093 (14)
C27	0.0184 (13)	0.0195 (14)	0.0249 (16)	0.0033 (11)	0.0063 (12)	0.0108 (13)
C28	0.0231 (14)	0.0242 (16)	0.0265 (17)	−0.0021 (12)	0.0050 (13)	0.0125 (14)
C29	0.041 (2)	0.0283 (18)	0.033 (2)	−0.0028 (15)	0.0063 (16)	0.0110 (17)
C30	0.049 (2)	0.037 (2)	0.030 (2)	−0.0120 (18)	0.0022 (18)	0.0100 (18)
C31	0.037 (2)	0.052 (3)	0.038 (2)	−0.0142 (18)	−0.0050 (17)	0.027 (2)
C32	0.0257 (16)	0.044 (2)	0.042 (2)	−0.0016 (15)	0.0002 (16)	0.027 (2)
C33	0.0223 (15)	0.0310 (18)	0.0323 (19)	−0.0002 (13)	0.0052 (14)	0.0167 (16)
C34	0.0271 (16)	0.0335 (19)	0.041 (2)	0.0092 (14)	0.0068 (15)	0.0174 (18)
F5	0.0495 (14)	0.0276 (12)	0.0478 (15)	−0.0026 (10)	0.0112 (12)	−0.0048 (11)
F6	0.0240 (10)	0.0421 (13)	0.0539 (15)	−0.0029 (9)	0.0100 (10)	0.0113 (12)
N5	0.0172 (11)	0.0211 (13)	0.0258 (14)	0.0053 (9)	0.0044 (10)	0.0131 (11)
N6	0.0332 (16)	0.0273 (16)	0.0378 (18)	−0.0041 (12)	0.0028 (14)	0.0082 (14)
C35	0.0220 (14)	0.0212 (15)	0.0263 (16)	0.0028 (11)	0.0032 (12)	0.0139 (14)
C36	0.0281 (16)	0.0230 (16)	0.0315 (18)	0.0040 (13)	0.0058 (14)	0.0098 (15)
C37	0.0369 (19)	0.0219 (17)	0.034 (2)	0.0007 (14)	0.0062 (16)	0.0063 (16)
C38	0.0230 (15)	0.0324 (18)	0.038 (2)	0.0016 (13)	0.0072 (14)	0.0162 (17)
C39	0.0247 (15)	0.0232 (16)	0.0270 (17)	0.0042 (12)	0.0065 (13)	0.0120 (14)
C40	0.0197 (14)	0.0234 (15)	0.0288 (17)	0.0038 (12)	0.0054 (13)	0.0124 (14)
C41	0.0215 (15)	0.0345 (19)	0.036 (2)	0.0012 (13)	0.0090 (14)	0.0140 (17)
C42	0.0262 (16)	0.038 (2)	0.038 (2)	0.0101 (14)	0.0149 (15)	0.0172 (18)
C43	0.0255 (15)	0.0276 (16)	0.0273 (17)	0.0091 (13)	0.0081 (13)	0.0157 (15)
C44	0.0226 (14)	0.0244 (16)	0.0284 (17)	0.0069 (12)	0.0090 (13)	0.0143 (14)
C45	0.0320 (16)	0.0278 (17)	0.0304 (18)	0.0093 (13)	0.0150 (14)	0.0146 (15)
C46	0.0383 (19)	0.037 (2)	0.036 (2)	0.0157 (16)	0.0150 (16)	0.0181 (17)
C47	0.054 (2)	0.039 (2)	0.042 (2)	0.0218 (19)	0.026 (2)	0.0185 (19)
C48	0.066 (3)	0.030 (2)	0.034 (2)	0.0056 (19)	0.024 (2)	0.0084 (18)
C49	0.040 (2)	0.037 (2)	0.0301 (19)	−0.0029 (16)	0.0104 (16)	0.0135 (17)
C50	0.0311 (17)	0.0330 (18)	0.0300 (18)	0.0037 (14)	0.0116 (15)	0.0164 (16)
C51	0.0281 (18)	0.056 (3)	0.036 (2)	0.0114 (17)	0.0069 (16)	0.019 (2)
C52	0.114 (8)	0.181 (12)	0.207 (14)	0.014 (8)	0.007 (8)	0.126 (11)
C53	0.183 (11)	0.123 (8)	0.161 (11)	0.051 (8)	0.048 (9)	0.100 (8)
C54	0.172 (10)	0.068 (6)	0.172 (12)	0.019 (6)	0.064 (8)	0.050 (7)
C55	0.070 (3)	0.071 (3)	0.071 (3)	0.020 (2)	0.023 (2)	0.035 (2)
Cl1	0.210 (2)	0.257 (3)	0.244 (3)	0.0489 (18)	0.0950 (19)	0.0951 (18)

### Geometric parameters (Å, º)

**Table:**

Ir1—C35	1.991 (3)	C29—C30	1.387 (6)
Ir1—N5	2.030 (3)	C29—H29	0.9500
Ir1—N1	2.056 (3)	C30—C31	1.372 (7)
Ir1—C1	2.061 (3)	C30—H30	0.9500
Ir1—C18	2.070 (3)	C31—C32	1.378 (6)
Ir1—N3	2.143 (3)	C31—H31	0.9500
F1—C3	1.349 (4)	C32—C33	1.396 (5)
F2—C4	1.359 (4)	C32—H32	0.9500
N1—C10	1.346 (4)	C33—C34	1.496 (5)
N1—C6	1.378 (4)	C34—H34A	0.9800
N2—C4	1.307 (5)	C34—H34B	0.9800
N2—C3	1.323 (5)	C34—H34C	0.9800
C1—C2	1.397 (5)	F5—C37	1.352 (4)
C1—C5	1.421 (4)	F6—C38	1.352 (4)
C2—C3	1.369 (5)	N5—C44	1.346 (4)
C2—H2	0.9500	N5—C40	1.367 (4)
C4—C5	1.389 (5)	N6—C38	1.310 (5)
C5—C6	1.453 (5)	N6—C37	1.322 (5)
C6—C7	1.393 (5)	C35—C36	1.405 (5)
C7—C8	1.378 (5)	C35—C39	1.420 (4)
C7—H7	0.9500	C36—C37	1.371 (5)
C8—C9	1.392 (5)	C36—H36	0.9500
C8—H8	0.9500	C38—C39	1.389 (5)
C9—C10	1.386 (5)	C39—C40	1.456 (5)
C9—C11	1.490 (5)	C40—C41	1.400 (5)
C10—H10	0.9500	C41—C42	1.372 (5)
C11—C12	1.392 (5)	C41—H41	0.9500
C11—C16	1.404 (6)	C42—C43	1.401 (5)
C12—C13	1.390 (7)	C42—H42	0.9500
C12—H12	0.9500	C43—C44	1.384 (5)
C13—C14	1.385 (8)	C43—C45	1.483 (5)
C13—H13	0.9500	C44—H44	0.9500
C14—C15	1.366 (7)	C45—C46	1.396 (5)
C14—H14	0.9500	C45—C50	1.413 (5)
C15—C16	1.401 (6)	C46—C47	1.384 (6)
C15—H15	0.9500	C46—H46	0.9500
C16—C17	1.502 (6)	C47—C48	1.367 (6)
C17—H17A	0.9800	C47—H47	0.9500
C17—H17B	0.9800	C48—C49	1.385 (6)
C17—H17C	0.9800	C48—H48	0.9500
F3—C20	1.345 (5)	C49—C50	1.386 (5)
F4—C21	1.346 (4)	C49—H49	0.9500
N3—C27	1.343 (4)	C50—C51	1.513 (5)
N3—C23	1.367 (4)	C51—H51A	0.9800
N4—C21	1.305 (5)	C51—H51B	0.9800
N4—C20	1.316 (5)	C51—H51C	0.9800
C18—C19	1.392 (5)	C52—C53	1.562 (14)
C18—C22	1.429 (4)	C52—H52A	0.9800
C19—C20	1.369 (5)	C52—H52B	0.9800
C19—H19	0.9500	C52—H52C	0.9800
C21—C22	1.394 (5)	C53—C54	1.400 (13)
C22—C23	1.460 (5)	C53—H53A	0.9900
C23—C24	1.395 (4)	C53—H53B	0.9900
C24—C25	1.377 (5)	C54—C54^i^	1.358 (18)
C24—H24	0.9500	C54—H54A	0.9900
C25—C26	1.396 (5)	C54—H54C	0.9900
C25—H25	0.9500	C55—Cl1^ii^	1.727 (3)
C26—C27	1.389 (4)	C55—Cl1	1.741 (3)
C26—C28	1.483 (5)	C55—H55A	0.9900
C27—H27	0.9500	C55—H55B	0.9900
C28—C29	1.395 (5)	Cl1—C55^ii^	1.727 (4)
C28—C33	1.410 (5)		
			
C35—Ir1—N5	80.32 (12)	C29—C28—C33	119.4 (3)
C35—Ir1—N1	98.62 (12)	C29—C28—C26	118.1 (3)
N5—Ir1—N1	174.15 (10)	C33—C28—C26	122.4 (3)
C35—Ir1—C1	92.12 (12)	C30—C29—C28	121.3 (4)
N5—Ir1—C1	94.75 (12)	C30—C29—H29	119.4
N1—Ir1—C1	79.51 (12)	C28—C29—H29	119.4
C35—Ir1—C18	94.00 (13)	C31—C30—C29	119.3 (4)
N5—Ir1—C18	88.72 (11)	C31—C30—H30	120.3
N1—Ir1—C18	97.10 (12)	C29—C30—H30	120.3
C1—Ir1—C18	173.39 (11)	C30—C31—C32	120.2 (4)
C35—Ir1—N3	170.98 (11)	C30—C31—H31	119.9
N5—Ir1—N3	94.39 (10)	C32—C31—H31	119.9
N1—Ir1—N3	87.37 (10)	C31—C32—C33	122.0 (4)
C1—Ir1—N3	95.61 (11)	C31—C32—H32	119.0
C18—Ir1—N3	78.49 (12)	C33—C32—H32	119.0
C10—N1—C6	119.6 (3)	C32—C33—C28	117.7 (3)
C10—N1—Ir1	123.6 (2)	C32—C33—C34	118.6 (3)
C6—N1—Ir1	116.6 (2)	C28—C33—C34	123.6 (3)
C4—N2—C3	114.9 (3)	C33—C34—H34A	109.5
C2—C1—C5	117.7 (3)	C33—C34—H34B	109.5
C2—C1—Ir1	129.4 (2)	H34A—C34—H34B	109.5
C5—C1—Ir1	112.8 (2)	C33—C34—H34C	109.5
C3—C2—C1	117.8 (3)	H34A—C34—H34C	109.5
C3—C2—H2	121.1	H34B—C34—H34C	109.5
C1—C2—H2	121.1	C44—N5—C40	120.0 (3)
N2—C3—F1	113.6 (3)	C44—N5—Ir1	122.9 (2)
N2—C3—C2	126.4 (4)	C40—N5—Ir1	117.0 (2)
F1—C3—C2	120.0 (4)	C38—N6—C37	114.1 (3)
N2—C4—F2	113.9 (3)	C36—C35—C39	116.7 (3)
N2—C4—C5	126.7 (3)	C36—C35—Ir1	128.7 (2)
F2—C4—C5	119.4 (3)	C39—C35—Ir1	114.4 (2)
C4—C5—C1	116.4 (3)	C37—C36—C35	117.6 (3)
C4—C5—C6	126.8 (3)	C37—C36—H36	121.2
C1—C5—C6	116.8 (3)	C35—C36—H36	121.2
N1—C6—C7	118.6 (3)	N6—C37—F5	113.9 (3)
N1—C6—C5	112.9 (3)	N6—C37—C36	127.4 (4)
C7—C6—C5	128.5 (3)	F5—C37—C36	118.6 (3)
C8—C7—C6	120.9 (3)	N6—C38—F6	113.8 (3)
C8—C7—H7	119.6	N6—C38—C39	126.6 (3)
C6—C7—H7	119.6	F6—C38—C39	119.5 (3)
C7—C8—C9	120.5 (3)	C38—C39—C35	117.4 (3)
C7—C8—H8	119.8	C38—C39—C40	127.0 (3)
C9—C8—H8	119.8	C35—C39—C40	115.6 (3)
C10—C9—C8	116.5 (3)	N5—C40—C41	118.9 (3)
C10—C9—C11	119.3 (3)	N5—C40—C39	112.5 (3)
C8—C9—C11	124.1 (3)	C41—C40—C39	128.6 (3)
N1—C10—C9	123.8 (3)	C42—C41—C40	120.1 (3)
N1—C10—H10	118.1	C42—C41—H41	120.0
C9—C10—H10	118.1	C40—C41—H41	120.0
C12—C11—C16	119.2 (4)	C41—C42—C43	121.2 (3)
C12—C11—C9	119.0 (4)	C41—C42—H42	119.4
C16—C11—C9	121.7 (3)	C43—C42—H42	119.4
C13—C12—C11	121.1 (5)	C44—C43—C42	115.9 (3)
C13—C12—H12	119.5	C44—C43—C45	121.8 (3)
C11—C12—H12	119.5	C42—C43—C45	122.3 (3)
C14—C13—C12	119.4 (4)	N5—C44—C43	123.7 (3)
C14—C13—H13	120.3	N5—C44—H44	118.1
C12—C13—H13	120.3	C43—C44—H44	118.1
C15—C14—C13	120.3 (5)	C46—C45—C50	119.1 (3)
C15—C14—H14	119.9	C46—C45—C43	118.1 (3)
C13—C14—H14	119.9	C50—C45—C43	122.7 (3)
C14—C15—C16	121.4 (5)	C47—C46—C45	121.4 (4)
C14—C15—H15	119.3	C47—C46—H46	119.3
C16—C15—H15	119.3	C45—C46—H46	119.3
C15—C16—C11	118.7 (4)	C48—C47—C46	119.7 (4)
C15—C16—C17	117.6 (4)	C48—C47—H47	120.2
C11—C16—C17	123.6 (4)	C46—C47—H47	120.2
C16—C17—H17A	109.5	C47—C48—C49	119.6 (4)
C16—C17—H17B	109.5	C47—C48—H48	120.2
H17A—C17—H17B	109.5	C49—C48—H48	120.2
C16—C17—H17C	109.5	C48—C49—C50	122.5 (4)
H17A—C17—H17C	109.5	C48—C49—H49	118.8
H17B—C17—H17C	109.5	C50—C49—H49	118.8
C27—N3—C23	119.4 (3)	C49—C50—C45	117.7 (3)
C27—N3—Ir1	124.7 (2)	C49—C50—C51	118.0 (3)
C23—N3—Ir1	115.9 (2)	C45—C50—C51	124.3 (3)
C21—N4—C20	114.6 (3)	C50—C51—H51A	109.5
C19—C18—C22	116.7 (3)	C50—C51—H51B	109.5
C19—C18—Ir1	129.1 (3)	H51A—C51—H51B	109.5
C22—C18—Ir1	114.1 (2)	C50—C51—H51C	109.5
C20—C19—C18	118.2 (3)	H51A—C51—H51C	109.5
C20—C19—H19	120.9	H51B—C51—H51C	109.5
C18—C19—H19	120.9	C53—C52—H52A	109.5
N4—C20—F3	114.1 (3)	C53—C52—H52B	109.5
N4—C20—C19	127.1 (4)	H52A—C52—H52B	109.5
F3—C20—C19	118.8 (4)	C53—C52—H52C	109.5
N4—C21—F4	113.4 (3)	H52A—C52—H52C	109.5
N4—C21—C22	126.5 (3)	H52B—C52—H52C	109.5
F4—C21—C22	120.1 (3)	C54—C53—C52	114.3 (9)
C21—C22—C18	116.9 (3)	C54—C53—H53A	108.7
C21—C22—C23	126.1 (3)	C52—C53—H53A	108.7
C18—C22—C23	117.0 (3)	C54—C53—H53B	108.7
N3—C23—C24	119.5 (3)	C52—C53—H53B	108.7
N3—C23—C22	114.0 (3)	H53A—C53—H53B	107.6
C24—C23—C22	126.4 (3)	C54^i^—C54—C53	120.0 (12)
C25—C24—C23	119.9 (3)	C54^i^—C54—H54A	107.3
C25—C24—H24	120.0	C53—C54—H54A	107.3
C23—C24—H24	120.0	C54^i^—C54—H54C	107.3
C24—C25—C26	121.1 (3)	C53—C54—H54C	107.3
C24—C25—H25	119.5	H54A—C54—H54C	106.9
C26—C25—H25	119.5	Cl1^ii^—C55—Cl1	113.6 (6)
C27—C26—C25	115.9 (3)	Cl1^ii^—C55—H55A	108.8
C27—C26—C28	122.8 (3)	Cl1—C55—H55A	108.8
C25—C26—C28	121.4 (3)	Cl1^ii^—C55—H55B	108.8
N3—C27—C26	124.1 (3)	Cl1—C55—H55B	108.8
N3—C27—H27	117.9	H55A—C55—H55B	107.7
C26—C27—H27	117.9	C55^ii^—Cl1—C55	66.4 (6)
			
C5—C1—C2—C3	1.5 (5)	C22—C23—C24—C25	−177.0 (3)
Ir1—C1—C2—C3	−176.6 (3)	C23—C24—C25—C26	0.1 (5)
C4—N2—C3—F1	178.4 (3)	C24—C25—C26—C27	−2.0 (5)
C4—N2—C3—C2	−1.9 (6)	C24—C25—C26—C28	178.1 (3)
C1—C2—C3—N2	0.9 (6)	C23—N3—C27—C26	−0.5 (5)
C1—C2—C3—F1	−179.5 (3)	Ir1—N3—C27—C26	179.8 (2)
C3—N2—C4—F2	−179.5 (3)	C25—C26—C27—N3	2.2 (5)
C3—N2—C4—C5	0.4 (5)	C28—C26—C27—N3	−177.8 (3)
N2—C4—C5—C1	1.9 (5)	C27—C26—C28—C29	−134.7 (4)
F2—C4—C5—C1	−178.2 (3)	C25—C26—C28—C29	45.2 (5)
N2—C4—C5—C6	−176.6 (3)	C27—C26—C28—C33	47.1 (5)
F2—C4—C5—C6	3.4 (5)	C25—C26—C28—C33	−133.0 (4)
C2—C1—C5—C4	−2.7 (4)	C33—C28—C29—C30	1.2 (6)
Ir1—C1—C5—C4	175.7 (2)	C26—C28—C29—C30	−177.0 (3)
C2—C1—C5—C6	175.8 (3)	C28—C29—C30—C31	−0.2 (6)
Ir1—C1—C5—C6	−5.7 (3)	C29—C30—C31—C32	−0.8 (6)
C10—N1—C6—C7	4.3 (4)	C30—C31—C32—C33	0.8 (6)
Ir1—N1—C6—C7	−170.7 (2)	C31—C32—C33—C28	0.2 (6)
C10—N1—C6—C5	−175.1 (3)	C31—C32—C33—C34	178.2 (4)
Ir1—N1—C6—C5	9.9 (3)	C29—C28—C33—C32	−1.2 (5)
C4—C5—C6—N1	175.8 (3)	C26—C28—C33—C32	176.9 (3)
C1—C5—C6—N1	−2.6 (4)	C29—C28—C33—C34	−179.0 (3)
C4—C5—C6—C7	−3.5 (5)	C26—C28—C33—C34	−0.9 (5)
C1—C5—C6—C7	178.1 (3)	C39—C35—C36—C37	−0.2 (5)
N1—C6—C7—C8	−2.5 (5)	Ir1—C35—C36—C37	−176.3 (3)
C5—C6—C7—C8	176.8 (3)	C38—N6—C37—F5	179.3 (3)
C6—C7—C8—C9	−1.4 (5)	C38—N6—C37—C36	−0.3 (6)
C7—C8—C9—C10	3.5 (5)	C35—C36—C37—N6	1.1 (6)
C7—C8—C9—C11	−179.8 (3)	C35—C36—C37—F5	−178.5 (3)
C6—N1—C10—C9	−2.2 (5)	C37—N6—C38—F6	178.9 (3)
Ir1—N1—C10—C9	172.4 (2)	C37—N6—C38—C39	−1.6 (6)
C8—C9—C10—N1	−1.7 (5)	N6—C38—C39—C35	2.4 (6)
C11—C9—C10—N1	−178.6 (3)	F6—C38—C39—C35	−178.1 (3)
C10—C9—C11—C12	126.0 (4)	N6—C38—C39—C40	−177.8 (4)
C8—C9—C11—C12	−50.7 (5)	F6—C38—C39—C40	1.8 (6)
C10—C9—C11—C16	−51.4 (5)	C36—C35—C39—C38	−1.3 (5)
C8—C9—C11—C16	131.9 (4)	Ir1—C35—C39—C38	175.4 (3)
C16—C11—C12—C13	1.8 (6)	C36—C35—C39—C40	178.8 (3)
C9—C11—C12—C13	−175.6 (4)	Ir1—C35—C39—C40	−4.5 (4)
C11—C12—C13—C14	−0.2 (7)	C44—N5—C40—C41	1.0 (5)
C12—C13—C14—C15	−1.5 (7)	Ir1—N5—C40—C41	177.7 (3)
C13—C14—C15—C16	1.5 (7)	C44—N5—C40—C39	−177.0 (3)
C14—C15—C16—C11	0.2 (6)	Ir1—N5—C40—C39	−0.3 (4)
C14—C15—C16—C17	−177.5 (4)	C38—C39—C40—N5	−176.8 (3)
C12—C11—C16—C15	−1.8 (5)	C35—C39—C40—N5	3.1 (4)
C9—C11—C16—C15	175.5 (3)	C38—C39—C40—C41	5.5 (6)
C12—C11—C16—C17	175.8 (4)	C35—C39—C40—C41	−174.6 (3)
C9—C11—C16—C17	−6.9 (6)	N5—C40—C41—C42	−2.6 (5)
C22—C18—C19—C20	−1.9 (5)	C39—C40—C41—C42	175.0 (4)
Ir1—C18—C19—C20	176.1 (3)	C40—C41—C42—C43	1.0 (6)
C21—N4—C20—F3	−177.2 (4)	C41—C42—C43—C44	2.1 (5)
C21—N4—C20—C19	1.7 (7)	C41—C42—C43—C45	−179.7 (3)
C18—C19—C20—N4	−0.6 (7)	C40—N5—C44—C43	2.3 (5)
C18—C19—C20—F3	178.3 (4)	Ir1—N5—C44—C43	−174.2 (2)
C20—N4—C21—F4	179.1 (4)	C42—C43—C44—N5	−3.8 (5)
C20—N4—C21—C22	−0.2 (6)	C45—C43—C44—N5	178.0 (3)
N4—C21—C22—C18	−2.2 (6)	C44—C43—C45—C46	139.1 (4)
F4—C21—C22—C18	178.5 (3)	C42—C43—C45—C46	−39.0 (5)
N4—C21—C22—C23	175.5 (4)	C44—C43—C45—C50	−43.0 (5)
F4—C21—C22—C23	−3.7 (6)	C42—C43—C45—C50	138.8 (4)
C19—C18—C22—C21	3.2 (5)	C50—C45—C46—C47	0.2 (6)
Ir1—C18—C22—C21	−175.2 (3)	C43—C45—C46—C47	178.2 (4)
C19—C18—C22—C23	−174.8 (3)	C45—C46—C47—C48	0.4 (6)
Ir1—C18—C22—C23	6.8 (4)	C46—C47—C48—C49	−0.6 (6)
C27—N3—C23—C24	−1.6 (5)	C47—C48—C49—C50	0.2 (6)
Ir1—N3—C23—C24	178.2 (2)	C48—C49—C50—C45	0.5 (6)
C27—N3—C23—C22	177.4 (3)	C48—C49—C50—C51	−176.9 (4)
Ir1—N3—C23—C22	−2.9 (3)	C46—C45—C50—C49	−0.7 (5)
C21—C22—C23—N3	179.7 (3)	C43—C45—C50—C49	−178.5 (3)
C18—C22—C23—N3	−2.5 (4)	C46—C45—C50—C51	176.5 (4)
C21—C22—C23—C24	−1.5 (6)	C43—C45—C50—C51	−1.3 (6)
C18—C22—C23—C24	176.3 (3)	C52—C53—C54—C54^i^	−176.7 (15)
N3—C23—C24—C25	1.8 (5)	Cl1^ii^—C55—Cl1—C55^ii^	0.000 (1)

Symmetry codes: (i) −*x*+1, −*y*+1, −*z*; (ii) −*x*+1, −*y*+2, −*z*+1.

### Hydrogen-bond geometry (Å, º)

*Cg*3, *Cg*4 and *Cg*6 are the centroids of the N4/C18–C21, N5/C40–C44, and C45–C50 rings, respectively.

**Table:**

*D*—H···*A*	*D*—H	H···*A*	*D*···*A*	*D*—H···*A*
C7—H7···F2	0.95	2.29	2.895 (5)	121
C24—H24···F4	0.95	2.22	2.851 (4)	123
C36—H36···F2^iii^	0.95	2.41	3.245 (4)	146
C41—H41···F6	0.95	2.32	2.917 (4)	121
C44—H44···N3	0.95	2.50	3.112 (4)	122
C46—H46···F3^ii^	0.95	2.50	3.067 (5)	119
C13—H13···*Cg*6^iv^	0.95	2.98	3.777 (7)	142
C55—H55*A*···*Cg*4^ii^	0.99	2.96	3.326 (9)	103
C55—H55*B*···*Cg*3^ii^	0.99	3.00	3.718 (10)	131
C55—H55*B*···*Cg*4^ii^	0.99	2.78	3.326 (10)	116

Symmetry codes: (ii) −*x*+1, −*y*+2, −*z*+1; (iii) −*x*+2, −*y*+1, −*z*+1; (iv) −*x*+2, −*y*+2, −*z*+1.
